# Impact of laryngopharyngeal reflux on subjective and objective voice assessments: a prospective study

**DOI:** 10.1186/s40463-016-0171-1

**Published:** 2016-11-08

**Authors:** Jérôme R. Lechien, Kathy Huet, Mohamad Khalife, Anne-Françoise Fourneau, Véronique Delvaux, Myriam Piccaluga, Bernard Harmegnies, Sven Saussez

**Affiliations:** 1 Laboratory of Anatomy and Cell Biology, Faculty of Medicine, UMONS Research Institute for Health Sciences and Technology, University of Mons (UMons), Avenue du Champ de mars, 6, B7000 Mons, Belgium; 2 Laboratory of Phonetics, Faculty of Psychology, Research Institute for Language Sciences and Technology, University of Mons (UMons), Mons, Belgium; 3 Department of Otorhinolaryngology and Head and Neck Surgery, RHMS Baudour, EpiCURA Hospital, Baudour, Belgium

**Keywords:** Laryngopharyngeal reflux, Reflux laryngitis, Voice, Subjective and objective assessment

## Abstract

**Background:**

Laryngopharyngeal reflux is a prevalent, not well-understood disease affecting a high proportion of patients who seek laryngology consultation. The objective of this prospective case series is to explore the subjective and objective voice modifications in Laryngopharyngeal reflux (LPR), especially the usefulness of acoustic parameters as treatment outcomes, and to better understand the pathophysiological mechanisms underlying the development of voice disorder.

**Methods:**

Forty-one patients with a reflux finding score (RFS) > 7 and a reflux symptom index (RSI) > 13 were enrolled and treated with pantoprazole 20 mg twice daily for three months. RSI, RFS, Voice Handicap Index (VHI), and Grade, Roughness, Breathiness, Asthenia, Strain and Instability (GRBASI) were assessed at baseline and after three months post-therapy. Acoustic parameters were measured by selecting the most stable interval of the vowel /a/. A study of correlations between acoustic measurements and laryngoscopic signs was conducted in patients with roughness. Statistical analysis was performed using Statistical Package for the Social Sciences (SPSS).

**Results:**

Significant improvement in RSI, RFS, VHI, jitter, percent jitter, relative average perturbation (RAP), shimmer, percent shimmer, and amplitude perturbation quotient (APQ) was found at 3 months of treatment (*p* < .05). A correlation analysis revealed significant correlations between the grade of dysphonia, breathiness, asthenia, instability and jitter, percent jitter, RAP, shimmer, percent shimmer and APQ. In dividing our cohort into two groups of patients according to the presence of roughness, shimmer, percent shimmer and APQ significantly improved in patients with roughness, but no positive correlation was found between acoustic parameters and laryngoscopic signs.

**Conclusion:**

Acoustic parameters can help to better understand voice disorders in LPR and can be used as treatment outcomes in patients with roughness.

## Background

Laryngopharyngeal reflux (LPR) is the back flow of gastric contents into the laryngopharynx where it comes in contact with the tissues of the upper aerodigestive tract [1]. It concerns 4 to 10 % of patients who seek Ear Nose Throat (ENT) consultation and 1 % of patients in primary care practice [2–4]. The most common symptoms reported are globus sensation (88 %), throat clearing (82 %), and voice disorders such as hoarseness (79 %) [5, 6]. Heartburn accounts for less than 40 % of cases, whereas esophagitis concerns only 25 % of LPR patients [7, 8]. The major etiologic factor for hoarseness of more than 3 months duration is LPR, with a prevalence of 55 to 79 % in hoarse patients [9–11]. In comparison with healthy subjects, LPR patients often reported abnormal subjective voice characteristics such as musculoskeletal tension, hard glottal attack, glottal fry, vocal forcing, forcing sensations, clamping, vocal fatigue, prolonged voice warm-up time, and restricted tone placement [12–14]. LPR signs include posterior commissure hypertrophy (89 %), vocal fold edema (79 %), hyperemia (79 %), and diffuse laryngeal edema (76 %)^5^. This clinical entity considerably affects patients’ quality of life by reducing the speaker’s communicative effectiveness [2, 15]. Specifically, LPR is related to 50 to 78 % of the population with voice complaints and 91 % of voice disorders in the elderly [16–18]. Based on these voice disorders, many authors have used acoustic parameters as outcomes of medical treatment efficacy in LPR patients or in LPR patients with hoarseness, but results are mixed and controversial among studies [19–21]. Undoubtedly, some observe improvements of some acoustic parameters values [20, 21], and others refute these results [22, 23]. These varied results do not help the understanding of the pathophysiological mechanisms underlying hoarseness in LPR patients. Specifically, some authors suggested that vocal fold edema may be the main sign responsible for irregular vocal fold vibration leading to hoarseness [13], whereas other suspected mechanisms include dryness, keratosis, thickening of the epithelium, ulcerative lesions and alterations of the Reinke space [24].

LPR disease has been the subject of several case-control studies, which have concluded that a significantly lower voice quality (subjective and objective assessments) in LPR patients compared to controls [25].

The aim of this study is i) to explore the subjective and objective voice evolutions in LPR disease (LPRD), ii) to assess the usefulness of acoustic parameters as treatment outcomes in the general and rough LPR populations, and iii) to better understand the pathophysiological mechanisms underlying the development of voice disorder.

## Methods

Forty-one adult outpatients who visited the ENT outpatient department of the Epicura Hospital (Belgium) with LPR-related symptoms (hoarseness, throat clearing, cough, globus pharyngeus, dysphagia, throat pain, excess throat mucus or postnasal drip, heartburn, etc.) since minimum 3 months were studied prospectively from September 2013 to March 2015. LPR diagnosis was performed by French versions of reflux symptom index (RSI) and reflux finding score (RFS), both initially developed by Belafsky et al. [26]. Indeed, even if the utilization of pH metry remains controversial, these authors have demonstrated that these thresholds (RSI > 13 and RFS > 7) were highly correlated with pathological pH monitoring (pH < 4) [26]. To be eligible as LPR patients in our study, patients must have presented an RSI score > 13 and an RFS score > 7. A physician (who did not know the results of the RSI) assessed the RFS score in a blind manner at baseline and after treatment. Patients were excluded if they met the following criteria: vocal overuse, neurological disease affecting voice, psychiatric illness, upper respiratory tract infections within the last month, an antacid treatment already started (i.e., proton pump inhibitor(s) (PPI(s)), gastroprokinetic, or antihistamine), previous history of cervical surgery or radiotherapy, laryngeal trauma, vocal cord paralysis/paresis, benign vocal fold lesions, pharyngolaryngeal malignancy, seasonal allergies, PPI hypersensitivity, untreated thyroid disease, prior antireflux surgery, or chemical exposure causing laryngitis. Moreover, active smokers, alcoholics and pregnant and lactating women were also excluded.

The study protocol was approved by the local ethical committee of the Epicura Hospital (n° A2014/001). After obtaining informed consent from each patient, they were treated with diet and lifestyle measures and twice-daily proton pump inhibitors (20 mg pantoprazole). Patients did not receive vocal hygiene teaching and they had not consulted a speech therapist. Both the patient and the physician have evaluated the respect of the diet advices after the treatment period using a scale ranging from 0 (recommendations not respected) to 10 (recommendations fully respected). At baseline and after 3 months of treatment, subjects completed questionnaires (RSI and voice handicap index (VHI)) and underwent videolaryngostroboscopy (RFS; StrobeLED - CLL-S1, Olympus Corporation, Hamburg, Germany) and voice recording by the same practitioner (JL). Among the perceptual voice items assessed by Grade, Roughness, Breathiness, Asthenia, Strain, Instability (GRBASI) score, roughness is often the most prevalent perceptual voice characteristic in LPR patients (without vocal abuse, etc.) [25]. At baseline, the main clinician (JL) performed a subjective evaluation of the perceptual roughness of the patients using GRBASI scale to classify the patients into two groups following the severity of the perceptual roughness: “patients without roughness” (absence or mild grade) and “patients with roughness” (moderate or severe grade). Moreover, an experienced physician performed the blinded assessment of the patient perceptual voice quality (with GRBASI) on the basis of the recordings. The physician did not know the time of the recording (pre and post-therapy). In regard to the voice analysis, subjects were instructed to produce the vowel /a/ three times, at modal phonation, for a time corresponding to the maximum phonation time to optimize the research of the most stable interval. Voice assessments were conducted in a sound-treated room with a high-quality microphone (Sony PCM-D50; New York, NY, USA) placed at a distance of 30 cm from the patient’s mouth. We treated the speech signal using MDVP® software (KayPentax®, Paragon Drive Montvale, NJ, USA) to measure Jitter percent (Jitt), Relative Average Perturbation (RAP), Pitch Perturbation Quotient (PPQ), Fundamental frequency variation (vF0), Shimmer percent (Shim), Amplitude Perturbation Quotient (APQ), Peak-to-Peak Amplitude Variation (vAm), and Noise Harmonic Ratio (NHR). Even if some acoustic parameters may be correlated, we made the choice to keep all parameters to evaluate their sensitivity in the assessment of the treatment effectiveness. The measurement of acoustic values at an interval of 1 s was considered the most stable by showing the lowest jitt, shim and NHR values. These measures were performed in the entire cohort and in patients with moderate and severe roughness following the physician assessment (GRBASI) and following the patient (RSI, first item and VHI total score > 20). An experienced physician performed a second assessment of GRBASI in a blind manner for the correlation study. A correlation study between the respect of treatment, the subcategories of RSI and RFS, blinded GRBASI items and acoustic parameters was conducted.

Statistical analysis was performed using the Statistical Package for the Social Sciences for Windows (SPSS version 22.0; IBM Corp. Armonk, NY). Changes in RSI, RFS, VHI, GRBASI scores were calculated using the Wilcoxon signed-rank test. The effect of treatment on acoustic parameters was also calculated using Wilcoxon signed-rank test, whereas correlations between diet respect, GRBASI, RSI, RFS and acoustic parameters were calculated using Pearson’s correlation test. A level of significance of .05 was adopted.

## Results

### Subject characteristics

From the 54 patients identified as candidates, 41 completed the study. There were 18 men (44 %) and 23 women (56 %). The mean age of subjects was 50 years (50 in the female subgroup (24–72), and 51 in the male subgroup (19–86)). The average body mass index of the participants was 26.64 kg/m^2^. There were no adverse reactions to the treatment. All patients respected the intake of PPIs. A lot of potential candidates were not recruited because they already were on PPI(s). The most common primary complaints concerned cough (*N* = 8, 19.51 %), globus sensation (*N* = 7, 17.07 %), odynophagia (*N* = 7, 17.07 %), and dysphonia (*N* = 6, 14.63 %). Other symptoms were found in less than 10 % of patients. When we focused on the complaints exhibited by RSI, throat clearing (*N* = 38, 92.68 %), dysphonia (*N* = 37, 90.24 %), mucous sensation/postnasal drip (*N* = 34, 82.93 %), and chest pain/heartburn/stomach disorder(s) (*N* = 33, 80.49 %) were the most prevalent symptoms.

### Clinical and subjective voice assessment evolution

**Fig. 1:**
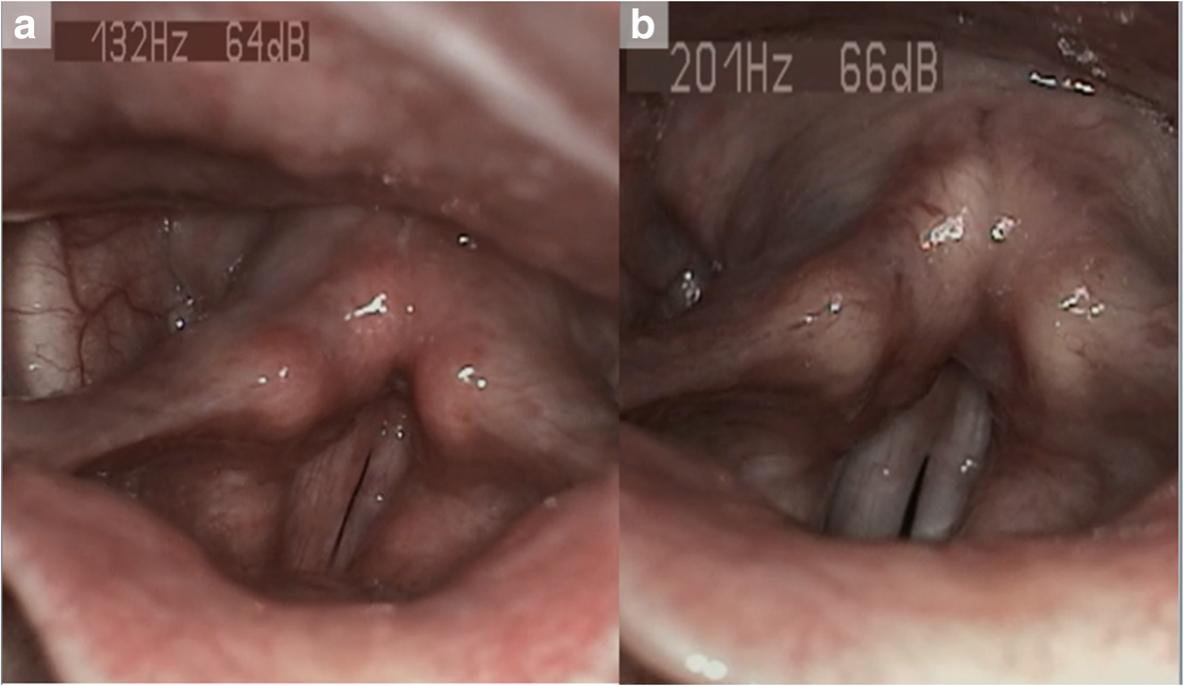
laryngological signs before and after treatment. The videostroboscopy at baseline (**a**) showed laryngeal and inter-arytenoid redness, posterior commissure hypertrophy, vocal folds irritation and pharyngolaryngeal edema suggesting laryngopharyngeal reflux disease. These signs improved after treatment (**b**)

In the first part of our study, we subjectively assessed the voice of our patients suffering from LPR before and after a three month treatment of pantoprazole (20 mg twice a day). Our subjective analysis comprised the RSI, RFS, VHI and GRBASI scores. The mean RSI for the pretreatment group was 22.98 ± 7.05, which was significantly higher than the mean RSI for the posttreatment group (8.02 ± 5.18) (Table 1). The mean value of RFS was 10.73 ± 2.24 in the pretreatment group and decreased significantly (4.61 ± 3.20) in the posttreatment group. Therefore, the clinical assessments demonstrated an important improvement characterized by a significant decrease in both RSI (*p* < 0.001) and RFS (*p* < 0.001) after 12 weeks of treatment (Table 1). Some clinical pictures of signs of LPR disease are available before and after treatment in Fig. 1.

**Table 1:** Pre- and posttreatment clinical and subjective voice assessments in LPR patients

Scales	pretreatment	posttreatment	Z	*p*-value*
RSI	22.98 ± 7.06	8.02 ± 5.18	−5.52	< 0.001
RFS	10.73 ± 2.24	4.61 ± 3.20	−5.44	< 0.001
VHI	18.07 ± 12.98	9.10 ± 8.93	−4.38	< 0.001
VHIe	3.54 ± 4.06	1.63 ± 2.90	−3.67	< 0.001
VHIp	9.58 ± 6.85	5.34 ± 5.13	−3.86	< 0.001
VHIf	4.90 ± 4.65	5.34 ± 5.13	−4.08	< 0.001
Blinded				
Grade	0.83 ± 0.67	0.80 ± 0.56	−0.23	0.819
Roughness	0.88 ± 0.71	0.76 ± 0.58	−1.04	0.297
Breathing	0.61 ± 0.74	0.56 ± 0.59	−0.43	0.670
Asthenia	0.44 ± 0.74	0.39 ± 0.67	−0.29	0.768
Strain	0.93 ± 0.76	0.98 ± 0.69	−0.36	0.721
Instability	0.98 ± 0.79	0.90 ± 0.77	−0.54	0.590

*Wilcoxon matched-pairs signed-ranks test (values and; *VHIf* Voice Handicap Index Functional, *VHIe* Voice Handicap Index Emotional, *VHIp* Voice Handicap Index Physic, *VHI* Voice Handicap Index

The mean VHI scoring assessed in the pretreatment group was 18.07 ± 12.98 and decreased significantly to 9.10 ± 8.93 after three months of treatment (*p* < 0.001). All subcategories scores (VHI, VHI emotional, VHI physic, VHI functional) decreased significantly after 3 months of treatment. According to Wilcoxon matched-pairs signed-ranks test, the perceptual voice quality of patients significantly improved in each GRBASI item after 3 months of therapy (Table 1). The blinded assessment of GRBASI did not reveal significant change after treatment.

### Acoustic parameters

The acoustic parameters in LPR patients before and after treatment are described in Table 2. Except PPQ, all values of the acoustic parameters measuring the short-term perturbation of the fundamental frequency i.e. Jitt, and RAP showed a significant improvement after treatment. PFR, the acoustic parameter measuring acoustic disturbance of F0 did not significantly improve after treatment. In regard to the acoustic parameters measuring the short-term perturbation of the intensity, Shim and APQ showed a significant improvement after treatment (Table 2). A study of correlations between RFS and RSI did not report a relevant correlation. In contrast, the potential correlations between GRBASI assessment and acoustic measurement revealed different significant correlations between the grade of dysphonia, breathiness, asthenia, instability and all relevant acoustic parameters (Table 3). The perception of strain was also significantly correlated only with Shim and APQ. The acoustic parameters of patients’ with/without roughness before and after treatment are described in Tables 4 and 5. All acoustic parameters did not improve after three months of treatment in the group of patients without roughness. In patients with roughness, Shim and APQ significantly improved after treatment (Table 5). Similar analyses were conducted in patients divided according to the presence of a pathological VHI score (VHI > 20) or the presence of voice disorder (RSI, first item), but few significant differences were found between the groups. In regard to potential correlations between the laryngoscopic signs (RFS), clinical symptoms (RSI) and acoustic parameters in rough patients, we did not find significant correlations between the main laryngoscopic signs, clinical symptoms and acoustic parameters. According to the Pearson correlation test, we found significant correlation between the respect of diet advices and the improvement of RSI score (z = -.420; *p* = .006).

**Table 2:** Pre- and posttreatment acoustic parameter assessment in LPR patients (mean ± inter-quartile values)

Acoustic Parameters	ULNR♯	pretreatment	posttreatment	Z	*p*-value*
STD	2.04	3.16 ± 2.38	2.59 ± 1.76	−1.34	0.180
vF0	1.10	1.92 ± 1.05	1.59 ± 0.92	−1.19	0.236
Jitt	0.61	1.42 ± 1.10	1.12 ± 1.10	−2.08	0.038
RAP	0.36	0.84 ± 0.65	0.67 ± 0.55	−2.01	0.044
PPQ	0.35	0.84 ± 0.66	0.68 ± 0.52	−1.94	0.053
PFR	2.17	3.10 ± 2.00	2.66 ± 1.00	−1.72	0.084
Shim	2.26	5.14 ± 2.90	4.12 ± 2.30	−2.73	0.006
APQ	1.69	4.35 ± 2.20	3.31 ± 1.82	−3.00	0.003
vAm	9.23	13.75 ± 9.60	13.30 ± 6.58	−0.84	0.403
NHR	0.12	0.14 ± 0.40	0.13 ± 0.03	−0.33	0.741

*Wilcoxon matched-pairs signed-ranks test. ULNR: Upper Limit of N Range, ♯ = based on the MDVP® norms

**Table 3:** Coefficient of correlation (coefficient and *p-value*) between voice subjective assessment (blinded GRBASI) and acoustic parameters

	G	*p*-value	R	*p*-value	B	*p*-value	A	*p*-value	S	*p*-value	I	*p*-value
Jitt	0.463	0.002	0.184	0.249	0.548	<0.001	0.505	0.001	0.295	0.061	0.430	0.005
RAP	0.454	0.003	0.190	0.234	0.530	<0.001	0.498	0.001	0.276	0.081	0.419	0.007
Shim	0.500	0.001	0.130	0.416	0.522	<0.001	0.397	0.010	0.417	0.007	0.365	0.019
APQ	0.494	0.001	0.159	0.320	0.463	0.002	0.405	0.009	0.438	0.004	0.401	0.009

The statistical analysis was provided using Pearson's correlation test; Grade (G), Roughness (R), Breathiness (B), Asthenia (A), Strain (S), Instability (I), Jitter percent (Jitt), Relative Average Perturbation (RAP), Shimmer percent (Shim), and Amplitude Perturbation Quotient (APQ)

**Table 4:** Pre- and posttreatment acoustic parameters assessment in LPR patient groups (patients with roughness vs. patients without roughness; mean and inter-quartile values)

		Patients without roughness (*n* = 26)			
A. Parameters	ULNR♯	pretreatment	posttreatment	Z	*p*-value*
STD	2.04	2.70 ± 1.60	2.49 ± 2.47	−0.85	0.395
vF0	1.10	1.69 ± 1.17	1.50 ± 1.18	−0.90	0.367
Jitt	0.61	1.34 ± 1.43	1.19 ± 1.37	−1.46	0.144
RAP	0.36	0.79 ± 0.71	0.71 ± 0.75	−1.26	0.209
PPQ	0.35	0.79 ± 0.65	0.71 ± 0.64	−1.33	0.182
PFR	2.17	2.77 ± 1.50	2.62 ± 1.00	−0.92	0.358
Shim	2.26	4.61 ± 1.92	4.17 ± 2.96	−1.05	0.292
APQ	1.69	3.81 ± 1.53	3.24 ± 2.88	−1.90	0.058
vAm	9.23	12.60 ± 11.93	13.56 ± 9.50	−0.16	0.869
NHR	0.12	0.13 ± 0.04	0.13 ± 0.03	−0.63	0.525

*Wilcoxon matched-pairs signed-ranks test; Acoustic parameters (A. parameters), Jitter percent (Jitt), Relative Average Perturbation (RAP), Pitch Perturbation Quotient (PPQ), Fundamental frequency variation (vF0), Shimmer percent (Shim), Amplitude Perturbation Quotient (APQ), Peak-to-Peak Amplitude Variation (vAm), and Noise Harmonic Ratio (NHR). ULNR: Upper Limit of N Range, ♯ = based on the MDVP norms

**Table 5:** Pre- and posttreatment acoustic parameters assessment in LPR patient groups (patients with roughness vs. patients without roughness; mean and inter-quartile values)

			Patients with roughness (*n* = 15)		
A. Parameters	ULNR♯	pretreatment	posttreatment	Z	*p*-value*
STD	2.04	3.94 ± 2.14	2.76 ± 1.48	−1.14	0.258
vF0	1.10	2.32 ± 1.13	1.74 ± 0.88	−0.97	0.334
Jitt	0.61	1.60 ± 1.17	1.02 ± 0.75	−1.36	0.173
RAP	0.36	0.92 ± 0.67	0.59 ± 0.50	−1.59	0.112
PPQ	0.35	0.91 ± 0.72	0.61 ± 0.40	−1.36	0.173
PFR	2.17	3.67 ± 2.00	2.73 ± 1.00	−1.48	0.358
Shim	2.26	6.06 ± 4.23	4.03 ± 2.78	−2.78	0.005
APQ	1.69	5.27 ± 3.85	3.44 ± 2.17	−2.33	0.020
vAm	9.23	15.73 ± 8.62	12.84 ± 4.14	−1.02	0.307
NHR	0.12	0.14 ± 0.04	0.13 ± 0.04	−1.25	0.211

*Wilcoxon matched-pairs signed-ranks test; Acoustic parameters (A. parameters), Jitter percent (Jitt), Relative Average Perturbation (RAP), Pitch Perturbation Quotient (PPQ), Fundamental frequency variation (vF0), Shimmer percent (Shim), Amplitude Perturbation Quotient (APQ), Peak-to-Peak Amplitude Variation (vAm), and Noise Harmonic Ratio (NHR). ULNR: Upper Limit of N Range, ♯ = based on the MDVP norms

## Discussion

Laryngopharyngeal reflux is a common disease that has been known as leading to chronic laryngitis and dysphonia. During the past two decades, a few studies have investigated the pathophysiological mechanisms underlying the development of LPR signs and symptoms, diagnosis, medical and surgical treatments. Although poorly and inaccurately documented and frequently observed by practitioners, voice disorders seem to be prevalent and may be disabling for patients. Thus, several case-control studies were conducted demonstrating significant differences in LPR patients concerning subjective (dysphonia and VHI) and objective (aerodynamic and acoustic) voice assessments in comparison with healthy subjects [25]. Given the limitations of the pH monitoring, Belafsky *et al.* developed RSI and RFS for both the diagnosis and follow-up of LPR signs and symptoms [16, 26]. These two scales are readily administered, highly reproducible, and exhibit excellent construct- and criterion-based validity [27]. We found that RSI and RFS improved significantly after 12 weeks of PPIs and diet behavioral changes. These findings are in accordance with previous studies that observed the decrease in RSI and RFS after PPI and diet treatment [20, 28–30]. Moreover, we observed a significant correlation between the respect of the diet advices and the improvement of the RSI score. This interesting finding strengthens the involvement of the respect of the diet in the enhancement of the clinical complaints. In contrast, we did not observe significant improvement of laryngoscopic signs, suspecting a kind of potential suggestion’s effect of the respect of the regimen on the symptoms improvement. We did not use pH metry given the many limitations. Firstly, it is well known that intermittent reflux may not occur during the test period. Thus, 3 episodes per week can be sufficient to generate LPR disease [31, 32]. These intermittent reflux episodes often lead to false negatives. Moreover, other false negatives or false positives may be secondary to the probe placement, movement or irritation [32]. Secondly, the normal values for the test could not be definitely established given the difficulty of carrying out this test in a large number of normal volunteers. Indeed, it seems that there would on average of 1.8 episodes per 24 h in healthy population [33] while another study reported LPR episodes in 52 % healthy subjects with a cut-off set to 2 episodes per day [34]. Other limitations (i.e., patient resistance, interpretation difficulties, patients rejection, cost, and equipment availability) limit the utilization of the pH metry and it is for these reasons that we decided to made the diagnosis using clinical scales. Among the LPR symptoms, many patients report voice disorders notably described through the VHI scale in LPRD [13]. In our study, we used the VHI scale to describe voice complaints and to indicate treatment efficiency. We found that total and subcategories of VHI scores significantly improved after treatment, confirming that VHI is an interesting tool to assess voice disorders in LPRD. These results corroborate those of Sereg-Bahar et al., which showed an improvement in VHI after 8 weeks of omeprazole therapy and dietary advice [35]. Siupsinkiene et al. also reported the interest to use VHI as an outcome of the efficacy of the PPI treatment in LPR patients [36]. At the exception of the study of Park et al., the perceptual voice quality assessments conducted in LPR studies were not blinded [13, 37–39]. The study of Park et al. showed a significant improvement of all GRBAS items after 3 months of treatment. These authors defined the significant improvement by the enhancement of ≥ 1 item(s) of the scale, which does not coincide with our statistical approach, limiting our literature comparison [39]. The low scores and the lack of significant improvement of the values of the GRBASI items could be related to a majority of mild and moderate LPR patient profiles composing our cohort. Thus, this hypothesis could highlight the interest for the acoustic measurements to assess the treatment efficiency. Indeed, it important to consider that subtle voice changes may be even more difficult to detect by the usual subjective assessment by the clinician or the patient him/herself. Therefore, many studies use various acoustic parameters to study the pathophysiology or to measure the effectiveness of treatment. In our study, many acoustic parameters (i.e., Jita, Jitt, RAP, Shim, ShdB, and APQ) improved after treatment in the entire cohort. In their prospective study, Jin et al. selected the most stable interval with the lowest jitter value [20]. They found significant changes in Jitt, Shim, and HNR at 3 months post-therapy. These findings were corroborated in our study only in regard to Jitt and Shim. Another study investigating the therapeutic benefit of lansoprazole or omeprazole plus speech therapy for 8 weeks provided no significant improvement in any of the acoustic characteristics studied (i.e., Jitt and Shim) [23]. Additionally, our results reported that acoustic parameters could be used primarily in rough patients. Indeed, after dividing our cohort into two patient groups according to the presence of roughness (assessed by the clinician), we observed a significant improvement in Shim, ShdB and APQ only in patients with roughness, and we had 3 more acoustic parameters in the total cohort. The acoustic parameters measuring the short-term perturbation of the fundamental frequency did not improve probably because of the reduction of statistical power due to the lower number of patients in this group. Shaw *et al.* showed that all rough patients with suspected LPR at baseline had significant changes in Jitt and Shim [19], whereas Hamdan found no significant modification in any of the acoustic parameter values studied (RAP, Shim, and NHR) after a short period of 4 weeks of PPI treatment [22]. The study by Shaw et al. reported that the utilization of acoustic measures is important, especially in rough LPR patients, but is less important in LPR patients without roughness [19]. Our results corroborate the fact that the voice quality (hoarseness, and especially roughness) perceived by the physician may suggest the utilization of acoustic parameters, such as an indicator of the healing of mucosal lesions and the treatment efficacy. Nevertheless, our results should be cautiously compared with the literature given the myriad of methods used to calculate the acoustic parameters. Indeed, the results of the acoustic measurements depend on the software used (and the algorithms underlying the calculation of acoustic measures), the type of vowel recorded, the duration of the analyzed segment, and the method of choice of the selected interval [25, 40]. Thus, the choice of the most stable interval of the vocal signal varies among studies. In our study, we adopted an objective method to select the most stable 1 s interval by selecting the portion with the lower values of jitter, shimmer, and NHR that represents an advantage of this study [41].

To better understand the pathophysiological mechanisms underlying the development of hoarseness, we conducted a correlation study in rough patients, which did not show a significant correlation between clinical symptoms (RSI), laryngoscopic signs (RFS) and acoustic measurements. These results stand in contrast to the study by Jin et al., which showed a significant positive correlation between Jitt and RSI [20]. Other previous studies did not report a correlation between signs and symptoms in LPR patients [42]. However, we found significant correlations between the grade of dysphonia, breathiness, asthenia, instability and the values of Jitt, RAP, Shim, and APQ. Some trials reported similar findings in other vocal diseases [43], but to the best of our knowledge, no LPR study has previously noted possible correlations between the values of acoustic parameters and GRBASI score. Strangely, we did not found the classical correlations between hoarseness or roughness and acoustic parameter as found in other diseases. A plausible explanation can be found by the representation of the GRBASI components by the experienced physician who heard a rough and breath voice that he considered, first and foremost, as breath voice. Similar findings have already been described [44]. Concerning the lack of correlation between signs and symptoms, several hypotheses can be identified. Firstly, our clinical experience makes us believe that patients develop their complaints in various ways. Some patients somatize more than others for the same complaint leading to differences in the final value of RSI. Secondly, we also observed in our clinical practice that some LPR signs causing clinical symptoms are not described in the RFS scale, such as hypertrophy of the lingual tonsils and vocal fold keratosis [45]. Regarding the development of roughness, some studies proposed that the most possible negative factors altering the periodicity of the vibration cycle and glottic closure would be slight edema of the vibratory margin of the vocal cords, which is caused by potentially noxious materials including gastric acid, pepsin and pancreatic enzyme irritation [13]. Other authors proposed that dryness (sticky laryngeal mucus), keratosis of the vibratory margin of the vocal folds, thickening of the epithelium, ulcerative lesions, granulomas and modifications of the Reinke space would form the basis of the alteration of the vibratory function of the vocal folds, especially in mild or moderate LPR patients [46]. Many of these conditions altering the mechanical and vibration characteristics of the vocal folds are not described in RFS and may lead to the development of roughness. In this study, we did not find a significant correlation between vocal fold edema, diffuse laryngeal edema, posterior commissure hypertrophy and subjective or objective voice assessments. Our cohort included a majority of patients with mild to moderate LPRD without severe signs of LPR (i.e., polypoid or/and severe vocal fold edema and/or granulomas) that could also explain our results. Finally, it is important to consider that genetic differences between individuals, particularly at the histological and biomolecular composition of the vocal folds, which may generate different local reactions to acid irritation characterized by various responses. Further histological studies are interesting to explore tissue modifications in LPR disease to precise some mechanisms. The main weakness of this study concerns the absence of a controlled group just treated by diet and behavioral changes. Indeed, to date, no study was interested to the impact of the diet vs the impact of the PPI(s) in the resolution of the voice problems in LPRD. Finally, the multiple statistical testings of this study were performed without a Bonferoni correction that may lead to an overstated significance.

## Conclusion

Our report highlights that changes in diet combined with pantoprazole twice daily neutralize the acidity responsible for the inflammation of the upper aerodigestive tract leading to an improvement of laryngeal symptoms, signs, perceptual voice disorders, and several acoustic parameters measuring the short-term perturbation of the fundamental frequency and the intensity, especially in rough patients. Thus, our correlation analyzis showed that the hoarseness (especially roughness) of the suspected LPR patients could be due to complex pathophysiological mechanisms and not simply to edema of the vocal folds such as reported previously [25]. In an obvious way, the healing of the vocal folds reported in suspected LPR patients could influence the voice, so that acoustic parameters would correlate with microscopic changes not always described in the RFS scale. These findings support the utilization of acoustic parameters (using an objective method to determine the most stable time interval) in the follow-up of LPR patients with hoarseness and to better understand vocal disorder development. Further randomized controlled trials with larger cohorts and objective acoustic methodological approaches are needed to confirm the role of each acoustic parameter in the follow-up of LPRD. Dryness and keratosis of the vocal folds could be systematically researched in our laryngological examination and also correlated to objective parameters.

## Abbreviations

A: Asthenia
APQ: Amplitude perturbation quotient
B: Breathing
ENT: Ear nose and throat
G: Grade
I: Instability
Jitt: Jitter percent
LPR: Laryngopharyngeal reflux
LPRD: Laryngopharyngeal reflux disease
NHR: Noise harmonic ratio
PPI: Proton pump inhibitors
PPQ: Pitch perturbation quotient
R: Roughness
RAP: Relative average perturbation
RFS: Reflux finding socre
RSI: Reflux symptom index
S: Strain
Shim: Shimmer percent
vAm: Peak-to-peak amplitude variation
vF0: Fundamental frequency variation
VHI: Voice handicap index
